# Comparative Analysis of Freeze-Dried *Pleurotus ostreatus* Mushroom Powders on Probiotic and Harmful Bacteria and Its Bioactive Compounds

**DOI:** 10.3390/jof11010001

**Published:** 2024-12-24

**Authors:** Gréta Törős, Áron Béni, Ferenc Peles, Gabriella Gulyás, József Prokisch

**Affiliations:** 1 Institute of Animal Science, Biotechnology and Nature Conservation, Faculty of Agricultural and Food Sciences and Environmental Management, University of Debrecen, Böszörményi Street 138, 4032 Debrecen, Hungary; gulyas@agr.unideb.hu (G.G.); jprokisch@agr.unideb.hu (J.P.); 2 Doctoral School of Animal Husbandry, Faculty of Agricultural and Food Sciences and Environmental Management, University of Debrecen, Böszörményi Street 138, 4032 Debrecen, Hungary; 3 Institute of Agricultural Chemistry and Soil Science, Faculty of Agricultural and Food Sciences and Environmental Management, University of Debrecen, Böszörményi Street 138, 4032 Debrecen, Hungary; beniaron@agr.unideb.hu; 4 Institute of Food Science, Faculty of Agricultural and Food Sciences and Environmental Management, University of Debrecen, Böszörményi Street 138, 4032 Debrecen, Hungary; pelesf@agr.unideb.hu

**Keywords:** prebiotics, antimicrobial agents, *Pleurotus ostreatus*, polysaccharide fraction, antioxidant activity, carbon nanodots

## Abstract

*Pleurotus ostreatus* (oyster mushroom) holds excellent promise worldwide, bringing several opportunities and augmenting the tool sets used in the biotechnology field, the food industry, and medicine. Our study explores the antimicrobial and probiotic growth stimulation benefits of freeze-dried *P. ostreatus* powders (OMP-TF, oyster mushroom powder from the total fresh sample; OMP-CSR, oyster mushroom powder from the cooked solid residue; OMP-CL, oyster mushroom powder from the cooked liquid), focusing on their bioactive compounds and associated activities. Our research examined polysaccharide fractions—specifically total glucans and α- and β-glucans—alongside secondary metabolites, including polyphenols and flavonoids, from freeze-dried mushroom powders. Additionally, carbon nanodots (CNDs) were also characterized. The growth inhibition was tested against *Escherichia coli* and *Staphylococcus epidermidis*, while the capacity for stimulating probiotic growth was evaluated using *Lactobacillus plantarum* and *Lactobacillus casei.* Evidence indicates that OMP-CL and OMP-CSR exhibit significant antimicrobial properties against *S. epidermidis* Gram-positive bacteria. OMP-CL notably promoted the growth of *L. casei*. OMP-CL, containing the most significant number of CNDs, has shown to be a valuable source for gut microbiota modulation, with its antimicrobial and probiotic-stimulating efficacy. However, further in vitro and in vivo studies should be performed to explore CNDs and their behavior in different biological systems.

## 1. Introduction

Mushrooms have become essential to modern diets thanks to their nutritional value and medicinal properties. This growing popularity has been primarily influenced by the rise in vegetarianism, which has significantly boosted mushroom consumption worldwide [1,2,3]. Among various edible mushroom types, *Pleurotus ostreatus* L., commonly known as the oyster mushroom, is one of the most widely consumed species due to its diverse culinary applications and health-promoting benefits [4,5].

*P. ostreatus* contains diverse bioactive compounds. However, polysaccharides, such as β-glucans and chitin, which contribute to its structural framework and functional benefits, are the most frequently studied components worldwide [6,7]. β-glucans are particularly noteworthy for their prebiotic, antimicrobial, and antioxidant properties, making them promising candidates for gut microbiota modulation. These polysaccharides selectively promote the growth of beneficial bacteria while suppressing harmful pathogens like *Escherichia coli*, thus establishing a strong connection between *P. ostreatus* and gut health improvement [8,9]. Furthermore, phenolic and flavonoid compounds from this mushroom are also essential, as they significantly reduce oxidative stress and support the production of antioxidant metabolites in the colon, contributing to gut health [2,10,11].

The bioactive compounds in *P. ostreatus* are sensitive to processing methods. Heat-induced reactions, such as the Maillard reaction (MR), can modify their activity and produce secondary compounds with additional health benefits [12,13]. Among these are melanoidins, which are macromolecules formed during MR that exhibit potent antioxidant, antimicrobial, and prebiotic properties [14,15]. Another byproduct of MR is carbon nanodots (CNDs), nanoscale carbon-based materials recognized for their photoluminescence, biocompatibility, and potential to enhance bioactivity [14,15,16]. Despite the promising benefits of melanoidins and CNDs, their precise interactions and contributions to health outcomes are still not fully understood [14]. CNDs can enhance the effectiveness of several bioactive compounds by helping deliver bioactive compounds into bacterial cells more effectively [17,18]. Furthermore, heat treatment can help break down the rigid walls of fungal cells and release bioactive compounds, such as phenolics, flavonoids [19,20], and polysaccharides, thereby boosting their functional benefits [21].

Our research explores the bioactive compounds that extend the antimicrobial and prebiotic properties of freeze-dried *P. ostreatus* mushroom powders. The polysaccharides (total glucans, α-glucans, and β-glucans), antioxidants (total flavonoids and polyphenols), and carbon nanodots formed during cooking were also investigated. Our study evaluates these compounds’ roles in gut microbiota modulation, including their capacity to stimulate probiotic bacteria (*Lactobacillus plantarum* and *Lactobacillus casei)*, suppress harmful bacteria (*Escherichia coli* and *Staphylococcus epidermidis*), and combat oxidative stress via radical scavenging activity (DPPH assay). By uncovering these mechanisms, the goal is to deepen the understanding of *P. ostreatus*’s therapeutic potential, particularly its role in promoting gut health and maintaining microbial balance.

## 2. Materials and Methods

### 2.1. Experimental Design for the Production and Characterization of Freeze-Dried P. ostreatus Mushroom

To synthesize mushroom powders, we utilized fresh *P. ostreatus* mushrooms sourced from PENNY Market Ltd. (Piactér brand, Hungary). After thoroughly washing and manually cutting the mushrooms into quarterers, the raw samples (LFP) were pre-frozen at −20 °C for 4 h in a stainless steel tray, ensuring optimal preparation for further processing.

The fresh mushroom materials were subjected to cooking at 90 °C in a sealed pressure cooker, ensuring a closed system to minimize liquid loss. The mushrooms were then placed in a drying oven (Venti-Line, VWR International Hungary Ltd., Debrecen, Hungary) and dried for 4 h. After drying, the pressure cooker was removed from the oven, and the cooked mushrooms were centrifuged at 1200 rpm for 10 min using a Hajdu C28.4B centrifuge with a capacity of 1 kg. Dairy filter paper (100 μm) was used to line the centrifuge equipment, ensuring efficient filtration. The filtered liquid was collected via an outlet tube and transferred into a designated vessel. The procedure was repeated three times, yielding an average liquid extraction rate of 57.19 ± 0.03% from the cooked sample. The average cooking loss, which was attributed to vapor release and evaporation during centrifugation, was calculated as 30.83 ± 2.81%; the procedure was conducted in three replicates and three different times to evaluate the reproductivity.

This method was previously described in our protocol [22], in which we assessed the incorporation of the produced mushroom liquid for *P. ostreatus* mycelia colonization. We have since enhanced the protocol with additional manufacturing steps (Points 1 and 2) for the optimum yield of the ground material, as follows:

(1) We utilized the liquid portion to create a dry powder; however, its viscous consistency initially complicated the freeze-drying process. To overcome this challenge, we strategically added a gelling agent, 2% (*m*/*v*) agar-agar (VWR International Hungary Ltd.). We heated it to 100 °C for 5 min, transforming it into a gel contained in a stainless steel tray. Once stabilized, the gel was perfectly prepared for freeze-drying.
(2) The cooked and stabilized liquid (filtrate), the cooked and centrifugated fruiting body (residue on the filter), and the raw mushroom samples (total fruiting body) were pre-frozen at −20 °C for 4 h without cooking. All pre-frozen samples were freeze-dried (Bionanoferm Ltd., Debrecen, Hungary). The freeze-drying process took 24 h at 40 °C. After freeze-drying, the samples were ground into fine powders.

The freeze-dried oyster mushroom powders (OMPs) were arranged in airtight containers and held in a dark area at temperatures below 25 °C until further analysis. As detailed in Table 1, each OMP was assigned a unique abbreviation for identification and easier management.

**Figure 1:**
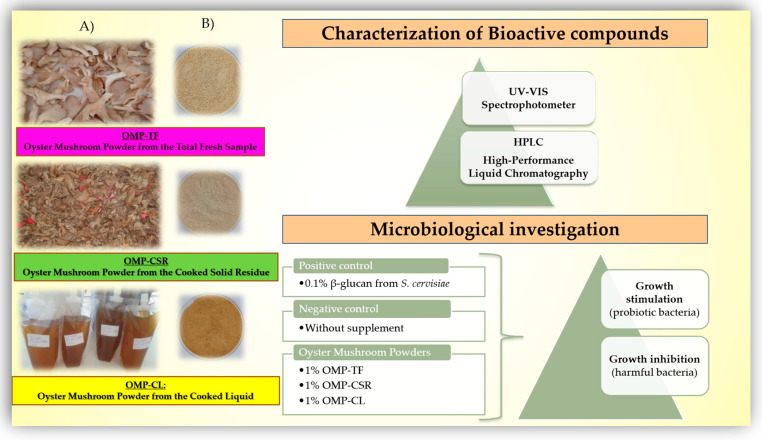
Summarization of the starter material for manufacturing oyster mushroom powders (OMPs) (right column, (**A**)) and final oyster mushroom powders OMPs (OMPs, (**B**)) (left column). **OMP-TF**, oyster mushroom powder from the total fresh sample; **OMP-CSR**, oyster mushroom powder from the cooked solid residue; **OMP-CL**, oyster mushroom powder from the cooked liquid; and the summarization of performed analysis. This figure includes each experiment performed and sample used for performing a microbiological investigation.

Figure 1 illustrates the mushroom samples before (Figure 1A) and after freeze-drying (Figure 1B) with the performed experiments. After production, the main active polysaccharide fractions, total glucan, α- and β-glucan content, and antioxidant compounds, namely, the total flavonoids and polyphenols, were studied using a UV-VIS spectrophotometer. CNDs and their molecular weights and fluorescence intensities were also investigated via high-performance liquid chromatography (HPLC). Radical scavenging activity (2,2-diphenyl-1-picrylhydrazyl) was tested. Microbiological investigations were performed to determine the suspected efficiency of OMPs on gut microbiota in vitro. The antimicrobial and prebiotic benefits were tested for different microorganisms, and then the prebiotic index was calculated.

### 2.2. Bioactive Compounds Presented in P. ostreatus Mushroom Powder

#### 2.2.1. Investigation of Glucan Content

α- and β-glucan and total glucan content have been determined in oyster mushroom samples using the Yeast Beta-Glucan Assay Kit (K-YBGL 02/21) developed by Megazyme (Bray, Ireland), following the method by McCleary and Draga (2016) [23].

The procedure involved hydrolyzing polysaccharides with exo-1,3-β-glucanase and β-glucosidase for total glucan and amyloglucosidase plus invertase for α-glucan. The glucan content was quantified by measuring absorbance at 510 nm using a UV-VIS spectrophotometer (Perkin Elmer), with the results expressed as *w*/*w*% via Megazyme’s Mega-CalcTM application. A β-glucan control was included to ensure result accuracy [23].

#### 2.2.2. Content of Phenolic and Flavonoid Compounds and Radical Scavenging Activity

Antioxidant agents and activity were recorded in 3 repetitions using a UV-VIS spectrophotometer (Perkin Elmer, Lambda, San Jose, CA, USA).

The total phenolic content (TPC) was quantified at 765 nm using the method described by Srivastava et al. (2024) [24], with minor modifications. A 0.1 g mushroom sample was prepared for measurement by homogenizing it in 10 mL of 80% methanol. The solution was filtered through a 0.45 μm hydrophilic PTFE syringe filter (Labex Ltd., New Delhi, India), and then 2.5 mL of 0.2 N Folin–Ciocalteu reagent was added. The samples were incubated at room temperature for 5 min. Next, 2.0 mL of a 7.5% (*m*/*v*) sodium carbonate (Na_2_CO_3_) solution was added, and the mixture was vortexed before being incubated in a dark environment at room temperature for 30 min. The control sample for the TPC assay was a blank solution consisting of 80% methanol. The standard for the calibration curve was gallic acid (VWR International Hungary Ltd.), which was constructed using known gallic acid concentrations (ranging from 0 to 100 µg/mL). The results were expressed as milligrams of gallic acid equivalents per gram of dry sample (mg GAE/g) [24].

The method of Wickramasinghe et al. (2023) [25], with minor modifications, was implemented to investigate total flavonoid content (TFC) at 510 nm. A 0.25 g sample of mushroom powder was combined with 1.25 mL of distilled water. To this, 75 µL of 5% (*m*/*v*) sodium nitrite (NaNO_2_) solution was added, followed by the addition of 150 µL of 10% (*m*/*v*) aluminum chloride hexahydrate (AlCl_3_·6H_2_O) solution after 6 min of incubation. After another 5 min, 0.5 mL of 1 M sodium hydroxide (NaOH) was added to the mixture, which was then adjusted to a final volume of 2.5 mL with distilled water and vortexed. The control sample for the TFC assay was a blank solution of distilled water. A calibration curve was prepared using known concentrations of catechin (VWR International Hungary Ltd.) ranging from 20 to 100 µg/mL). The flavonoid content was calculated on this calibration curve and expressed as milligrams of catechin equivalents per gram of dry sample (mg CE/g) [25].

The radical scavenging activity of 2,2-diphenyl-1-picrylhydrazyl (DPPH) was measured following the method described by Ahmad et al. (2014) [26], with minor modification at an absorbance of 517 nm. Each OMP extract’s radical scavenging potential was evaluated using the stable DPPH radical. The test extracts and the DPPH solution (VWR International Hungary Ltd.) were prepared in ethanol to maintain solvent consistency. To prepare the DPPH stock solution, 1.25 mg of accurately weighed DPPH powder was dissolved in 20 mL of ethanol (4× concentration). For the assay, a spectrophotometer cuvette combined 1.0 mL of the sample solution with 2.0 mL of the DPPH solution. The mixtures were incubated in the dark at room temperature for approximately 30 min. After incubation, the absorbance of each solution was recorded. The percentage of DPPH discoloration represented radical scavenging activity, and the results were expressed as the percentage of DPPH scavenging capacity (SC% for dry matter) [26].

#### 2.2.3. Size, Molecular Mass, and Final Concentrations of CNDs Presented in Freeze-Dried *P. ostreatus* Mushroom Powders (OMPs)

Our HPLC measurements to investigate carbon nanodots were performed according to the guidelines of Nguyen et al. (2024), which involve the possible formation of CNDs after the heat processing of food products and exploring heat-induced reactions [27].

To measure the possible formation of mushroom-based carbon nanodots at applied technology (Section 2.1) in the final products, freeze-dried OMPs made from low-temperature, long-cooked (4 h, 90 °C), and uncooked fresh samples have also been tested.

HPLC measurements included size exclusion chromatography and were applied to study the size and molecular mass presented in OMPs. Separations were performed on an Agilent AdvanceBio SEC column (300 Å; 4.6 × 300 mm × 2.7 μm) using an isocratic elution system. Distilled water was used to dilute (100×) water-soluble bioactive compounds (CNDs) in OMPs and filtered through a 0.45 μm hydrophilic PTFE syringe filter (Labex Ltd.).

The fluorescent detector (Shimadzu RF-20A, Kyoto, Japan) is connected to the HPLC (ECOM, ECS05, Chrastany, Czech Republic) system. The mobile phase (20–80% of the combination of acetonitrile and water) ran with a 0.7 mL/min flow rate. A total of 5 μL of the sample was injected into the system. Furthermore, 370 nm (excitation) and 460 nm (emission) wavelengths were applied to measure CNDs in cooked, uncooked, and freeze-dried mushroom powders. The system was calibrated using two peptide standard mixtures to determine the molecular mass. The first, a Bio-Rad gel filtration standard (Bio-Rad, Hercules, CA, USA), included a molecular weight range from 1350 to 670,000 Da comprising thyroglobulin, γ-globulin, ovalbumin, myoglobin, and vitamin B_12_. The second standard, sourced from Merck (Rahway, NJ, USA), contained peptides with specific molecular weights: Gly-Tyr (238.2 Da), Val-Tyr-Val (379.5 Da), methionine enkephalin (573.7 Da), leucine enkephalin (555.6 Da), and angiotensin II (1046.2 Da). These standards enabled the precise calibration and molecular mass identification of CNDs in the samples [27].

The carbon nanodot standard from glycin and dextrose prepared by Nguyen et al. (2024) was used to identify carbon nanodots in mushroom powders. The calibration series was made with 4 points from pure carbon nanodot powder dissolved in distilled water (from 0.01 g/mL to 0.00001 g/mL). A calibration curve with a known concentration was developed to obtain the final concentration of carbon nanodots in OMPs.

### 2.3. In Vitro Study of the Effectiveness of OMPs for the Beneficial Modulation of Gut Microbiota

#### 2.3.1. Test Microorganisms

Probiotic bacteria, namely, *Lactobacillus plantarum* (B.023391) and *Lactobacillus casei* (B.01526), were used to study the growth stimulation efficacy. Gram-negative (*Escherichia coli*, B.02357) and Gram-positive *(Staphylococcus epidermidis*, B.02055) bacteria strains were used to investigate the antimicrobial effect. All strains were obtained from the Reference collection of the Hungarian University of Agriculture and Life Sciences. Bacteria were stored at −80 °C (ultra-deep freezer) in a dehydrated form until later use, and then pure cultures were rehydrated to obtain viable cultures.

#### 2.3.2. Microbiological Assay Setup and Preparation of Starter Cultures

This study was conducted in the Microbiology Laboratory of the Food Science Institute, University of Debrecen, employing a microbiological method to evaluate the probiotic growth stimulation and antimicrobial effects of oyster mushroom powders (OMPs-OMP-TF, OMP-CSR, and OMP-CL).

Starter cultures were prepared by inoculating sterile nutrient broths (MRS, BioLab Zrt., Budapest, Hungary) with rehydrated bacterial colonies (Section 2.3.1) collected from solid MRSA agar using sterile cell scrapers. After vortexing, the cultures were incubated overnight at 37 °C.

#### 2.3.3. Treatment and Experimental Design

Microbiological experiments were conducted with five treatment groups to evaluate the functional properties of oyster mushroom powders (OMPs); 3 OMP groups and 2 control groups (positive and negative) were tested. Applied concentrations of OMPs and positive controls were determined according to the literature data from animal experiments [28] and designated with the following abbreviations:

(1) **1% OMP-TF** → 1% (*w*/*v*) of oyster mushroom powder from the total fresh sample.
(2) **1% OMP-CSR** → 1% (*w*/*v*) of oyster mushroom powder from the cooked solid residue.
(3) **1% OMP-CL** → 1% (*w*/*v*) of oyster mushroom powder from the cooked liquid.
(4) **0.1% PC** → 0.1% (*w*/*v*) commercially available β-glucans extracted from *Saccharomyces cerevisiae* (Medinvest Hungary Ltd., Kecskemét, Hungary), positive control.
(5) **NC** → Without added supplementation, a negative control means no treatment.

Each treatment received 1 cm^3^ of MRS broth containing pure bacterial cultures, which was added to 9 cm^3^ of sterile MRS broth supplemented with the respective treatment. Supplements were incorporated directly without additional solvents. The final volume in each tube was 10 cm^3^. After vortexing (60 s), the tubes were incubated at 37 °C for 6 h.

#### 2.3.4. Serial Dilution, Plating, and Incubation

For bacterial quantification, sterile peptone water (VWR International Hungary Ltd.) was prepared by dissolving 1.0 g of peptone and 8.5 g of sodium chloride in 1000 mL of distilled water. Serial dilutions were made up to 10^−8^ by transferring 1 cm^3^ of bacterial suspension from each treatment into 9 cm^3^ of sterile peptone water and vortexing. Bacterial isolation was performed using specific growth media, like MRSA medium (BioLab Zrt.) for *L. plantarum* and *L. casei* and PCA medium (BioLab Zrt.) for *E. coli* and *S. epidermidis.*

Two plating methods were employed for *L. plantarum*, *L. casei*, and *S. epidermidis*, which involved spreading 0.1 cm^3^ of the 10^−6^, 10^−7^, and 10^−8^ dilution levels onto the surface of solid media in Petri dishes.

While the casting method was applied for *E. coli*, 1 cm^3^ of the same dilution levels was added to empty Petri dishes. PCA medium was poured into the dishes and gently mixed to homogenize.

Plates were incubated at 37 °C for 48 ± 2 h. Colony-forming units (CFU) were enumerated and expressed as CFU/g bacteria.

### 2.4. Statistical Analysis

The significance of differences (*p* < 0.05) was compared across the tested groups for various bioactive compounds. Statistical analyses were based on data distribution: one-way ANOVA with Tukey’s HSD for normal data and Kruskal–Wallis with Dunn’s post hoc for non-normal data. All results are presented as means ± SD in tables and graphs generated using Microsoft Office Excel. Statistical tests are performed with SPSS statistical software 29.0. Distinct letters (a, b, and c) in tables and figures signify significant differences (*p* < 0.05).

## 3. Results

### 3.1. Polysaccharide Fractions (Glucans)

**Figure 2:**
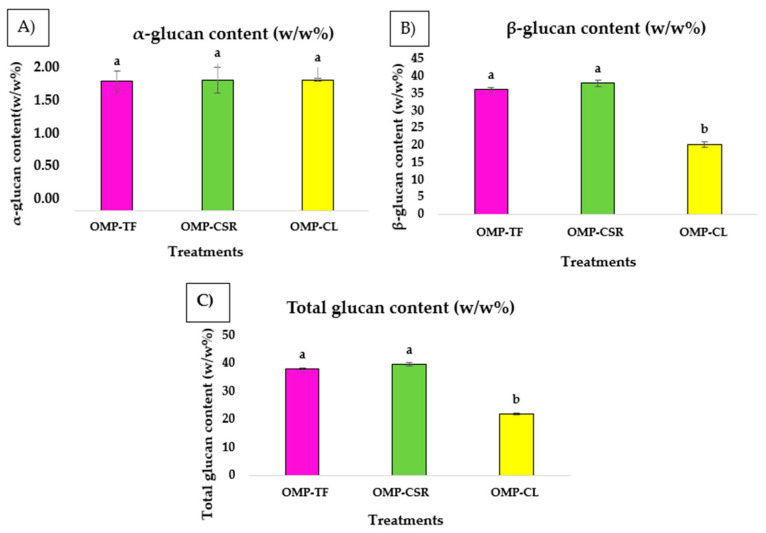
(**A**) α- and (**B**) β-glucan (*w*/*w*%); (**C**) total glucan = α + β-glucan (*w*/*w*%). The results are expressed as a volume percentage (n = 6 per mushroom sample). **OMP-TF**, oyster mushroom powder from the total fresh sample; **OMP-CSR**, oyster mushroom powder from the cooked solid residue; **OMP-CL**, oyster mushroom powder from the cooked liquid. Values are presented as means ± SD, and significant differences (*p* < 0.05) within the columns (OMP-TF, OMP-CSR, OMP-CL) are indicated by different (a and b) letters.

The non-parametric Kruskal–Wallis test performed on model products (OMP-TF, OMP-CSR, and OMP-CL) revealed significant differences in total glucan (Figure 2C) and β-glucan (Figure 2B) content between the OMP-CSR and OMP-CL groups (*p* < 0.05), as indicated by differing letters in Figure 2. According to the results, it can be seen that OMP-CL (20.27 ± 0.58 *w*/*w*%) contains significantly lower β-glucan content than OMP-CSR (37.95 ± 0.78 *w*/*w*%), followed by OMP-TF (36.25 ± 0.50 *w*/*w*%). The OMP-CSR sample has the highest total glucan value (39.17 ± 0.95 *w*/*w*%) by far. This is significantly more than the OMP-CL sample, which has a total glucan content of 22.06 ± 0.51 *w*/*w*%. This substantial difference clearly shows that there can be significant variations in this parameter between different OMPs. Furthermore, the ANOVA test found no significant differences (*p* > 0.05) in α-glucan (Figure 2A) content across the treatment groups: OMP-TF (1.70 ± 0.14 *w*/*w*%), OMP-CSR (1.72 ± 0.02 *w*/*w*%) and OMP-CL (1.72 ± 0.02 *w*/*w*%).

### 3.2. Production of CNDs and Their Properties (Molecular Mass and Size) at Different Fractions

**Figure 3:**
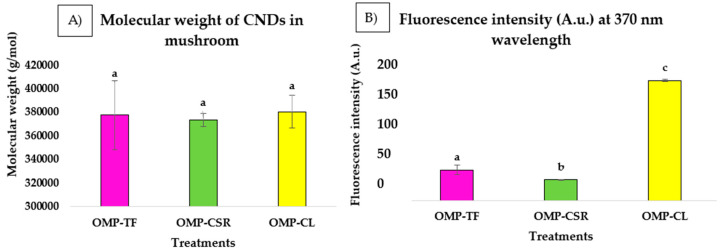
(**A**) Comparison of the molecular weight (g/mol) of OMPs and (**B**) fluorescence intensity (A.u.) at the ionization wavelength of 370 nm (mean ± SD). **OMP-TF**, oyster mushroom powder from the total fresh sample; **OMP-CSR**, oyster mushroom powder from the cooked solid residue; **OMP-CL**, oyster mushroom powder from the cooked liquid. Significant differences (*p* < 0.05) described with different alphabets (a, b, and c).

Figure 3 below illustrates the results of the HPLC analysis, including the molecular weight (Figure 3A) and peak areas (Figure 3B), which have been integrated from the chromatogram.

**Figure 4:**
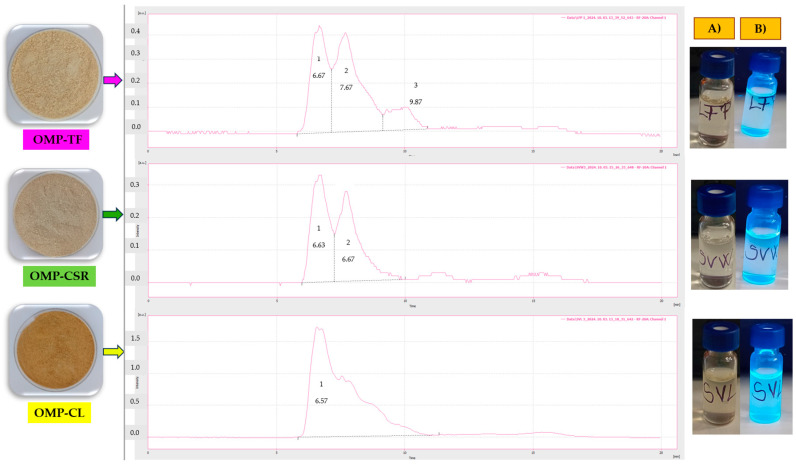
HPLC size exclusion chromatogram of OMPs (LFP, SVW, and SVL) where the (**A**) column presents a picture of diluted and filtered samples affected by visible light and (**B**) presents samples affected by the UV lamp. **OMP-TF**, oyster mushroom powder from the total fresh sample; **OMP-CSR**, oyster mushroom powder from the cooked solid residue; **OMP-CL**, oyster mushroom powder from the cooked liquid.

The chromatogram revealed the results of the analysis of OMP-TF, OMP-CSR, and OMP-CL (Figure 4). The ANOVA test revealed that CND is consistent in molecular weight (g/mol) in OMPs (Figure 4A). OMP-CL resulted in the highest molecular weight (g/mol), followed by OMP-TF, with 377,702 ± 29,144 g/mol. The lowest is shown to be the OMP-CSR, with (373,862 ± 5608 g/mol).

There is a significant difference in emitted fluorescence intensity (A.u.) (Figure 4B). It can be seen that all of the samples differed significantly. The OMP-CSR sample showed the lowest intensity (30.48 ± 0.46 A.u.), followed by OMP-TF (44.96 ± 7.20 A.u.). The OMP-CL sample exhibited the highest intensity (176.72 ± 2.00 A.u.). High-fluorescence properties are also visible in Figure 4B (affected by the UV lamp), compared with Figure 4A (without being affected by the UV lamp).

**Figure 5:**
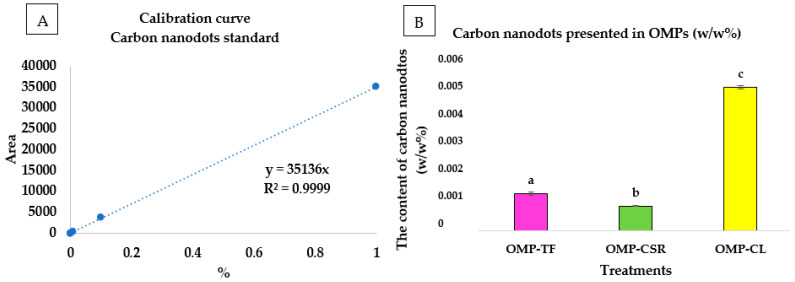
Calibration curve (**A**) and results in *w*/*w*% (**B**) of investigating the content of carbon nanodots. **OMP-TF**, oyster mushroom powder from the total fresh sample; **OMP-CSR**, oyster mushroom powder from the cooked solid residue; **OMP-CL**, oyster mushroom powder from the cooked liquid. Significant differences (*p* < 0.05) in Fig5B described with different alphabets (a, b, and c).

The statistical analysis demonstrated a clear and consistent trend in the fluorescence intensity measurements of carbon nanodots, as described in Figure 5, with the statistical significance confirmed (*p* < 0.05). These results (Figure 5B) were obtained following the development of a calibration curve (Figure 5A), which was constructed using a standard calibration series of carbon nanodots. It can be seen that the OMP-CSR sample showed the lowest amount of carbon nanodots (0.00086 ± 0.00003 *w*/*w*%), followed by OMP-TF (0.00131 ± 0.00007 *w*/*w*%), and the highest amount was achieved by OMP-CL (0.00504 ± 0.00005 *w*/*w*%)

### 3.3. Investigation of Antioxidants and 11,1-Diphenyl-2-Picryl-Hydrazyl (DPPH)

A Kruskal–Wallis test was performed to assess the differences in total polyphenol content (TPC), flavonoid levels (TFC), and antioxidant activity (DPPH) across three different *Pleurotus ostreatus* mushroom powders (**OMP-TF**, **OMP-CSR**, and **OMP-CL**).

**Figure 6:**
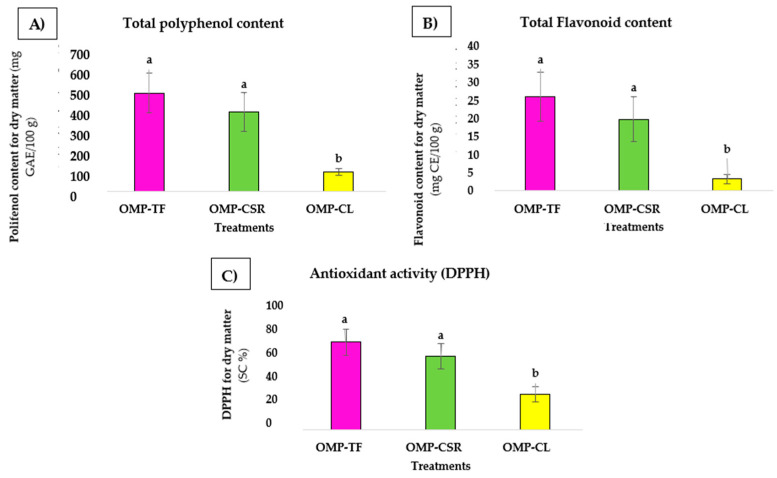
(**A**) Total polyphenol, (**B**) total flavonoid content, and (**C**) antioxidant activity (DPPH). **OMP-TF**, oyster mushroom powder from the total fresh sample; **OMP-CSR**, oyster mushroom powder from the cooked solid residue; **OMP-CL**, oyster mushroom powder from the cooked liquid. The results are expressed as a volume percentage (n = 6 per sample). Values are presented as means ± SD—significant differences in different letters (a, b).

The results revealed statistically significant differences, with the OMP-CL group differing significantly from OMP-CSR and OMP-TF (*p* < 0.05), as indicated by the distinct letter groupings in Figure 5. For each case, the TPC (Figure 6A), TFC (Figure 6B), and DPPH (Figure 6C) can be seen in Figure 6.

For antioxidant activity, as measured using the DPPH radical scavenging capacity, the OMP-CL (27.48 ± 5.82 SC%) displayed significantly lower activity compared to OMP-TF (67.85 ± 10.36 SC%) and OMP-CSR (57.09 ± 9.98 SC%). The trends were similar for TPC and TFC content. OMP-CL (98.86 ± 17.23 mg GAE/100 g) exhibited significantly lower polyphenol levels compared to both OMP-TF (489.42 ± 97.95 mg GAE/100 g) and OMP-CSR (396.23 ± 95.68 mg GAE/100 g). Furthermore, OMP-CL (3.32 ± 1.31 mg CE/100 g) contained significantly lower flavonoid levels compared to OMP-TF (26.66 ± 6.97 mg CE/100 g) and OMP-CSR (20.31 ± 6.43 mg CE/100 g).

### 3.4. Probiotic Growth Stimulation of OMPs

**Figure 7:**
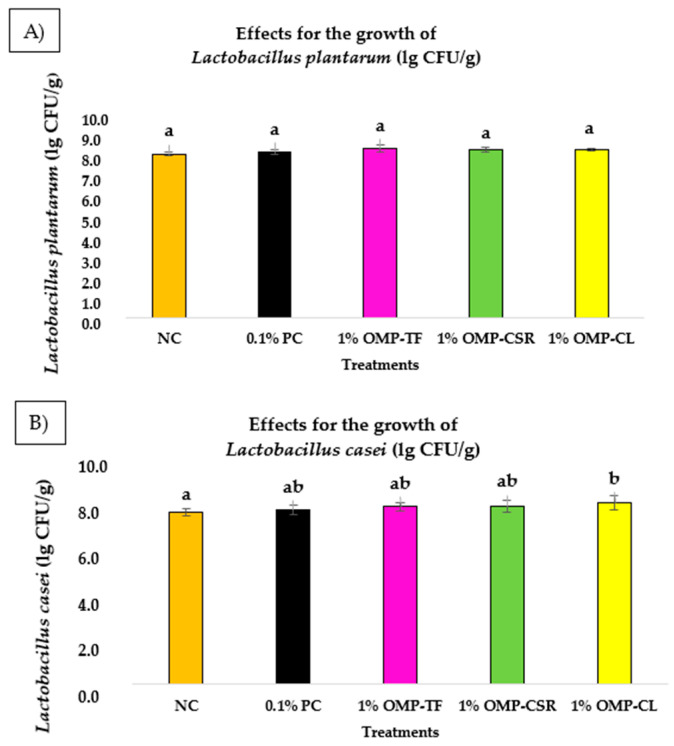
(**A**) Testing probiotic growth stimulation (*L. plantarum*) → (MRS liquid broth+1% OMP-TF; 1% OMP-CSR; 1% OMP-CL) compared with control groups: (1) *L. plantarum* + NC (MRS without added supplementation) and (2) 0.1% PC (MRS + 0.1% (*v*/*v*) β-glucans extracted from *S. cerevisiae* (Medinvest Hungary Ltd.)). (**B**) *L. casei* probiotic bacteria (same treatments as (**A**)). Results are conveyed as lg CFU/g, with values marked as lg means ± SD (n = 6 for each sample). Significant differences (*p* < 0.05) in Fig7B are described with different alphabets (a, b).

According to the one-way ANOVA test, significant differences (*p* < 0.05) were not observed among all the groups, indicating the influence of individual treatments on the growth of *Lactobacillus* spp. on MRS agar media, despite visible differences between groups. The bar chart below (Figure 7) illustrates the average growth of tested probiotics (*L. plantarum* (Figure 7A) and *L. casei* (Figure 7B) of freeze-dried oyster mushroom powders (OMPs) (measured in CFU/g) under different treatments.

The average value for the growth of *L. plantarum* bacteria in the negative control group (NC) was the lowest, at 8.66 ± 0.07 lg CFU/g, followed by 0.1% β-glucan at 8.76 ± 0.13 lg CFU/g (0.1% PC). In the cooked samples, 1% OMP-CSR presented 8.86 ± 0.12 lg CFU/g, which was lower than 1% OMP-CL at 8.90 ± 0.08 lg CFU/g. It can be stated that 1% OMP-TF exhibited the highest growth of *L. plantarum* at 8.97 ± 0.20 lg CFU/g. However, the results for *L. plantarum* did not differ significantly (*p* > 0.05) between the tested groups.

Compared to the control group, the lowest average growth of *L. casei* was observed at 1% OMP-TF, followed by the negative control (NC), which had an average of 7.88 ± 0.16 lg CFU/g. With the addition of 0.1% β-glucan (positive control, 0.1% PC), the average was slightly higher at 7.97 ± 0.20 lg CFU/g. However, other OMP treatments showed moderate results for *L. casei*, with 1% OMP-TF at 8.12 ± 0.19 lg CFU/g and 1% OMP-CSR at 8.13 ± 0.26 lg CFU/g. The highest average for *L. casei* was exhibited by 1% OMP-CL, with 8.23 ± 0.32 lg CFU/g, which was significantly higher than that of the negative control (*p* < 0.05).

### 3.5. Antimicrobial Activity of OMPs

The antimicrobial activity (in vitro) of the OMPs was estimated as a marker for their biological potential and gave us information before we tested the products’ therapeutic effects.

**Figure 8:**
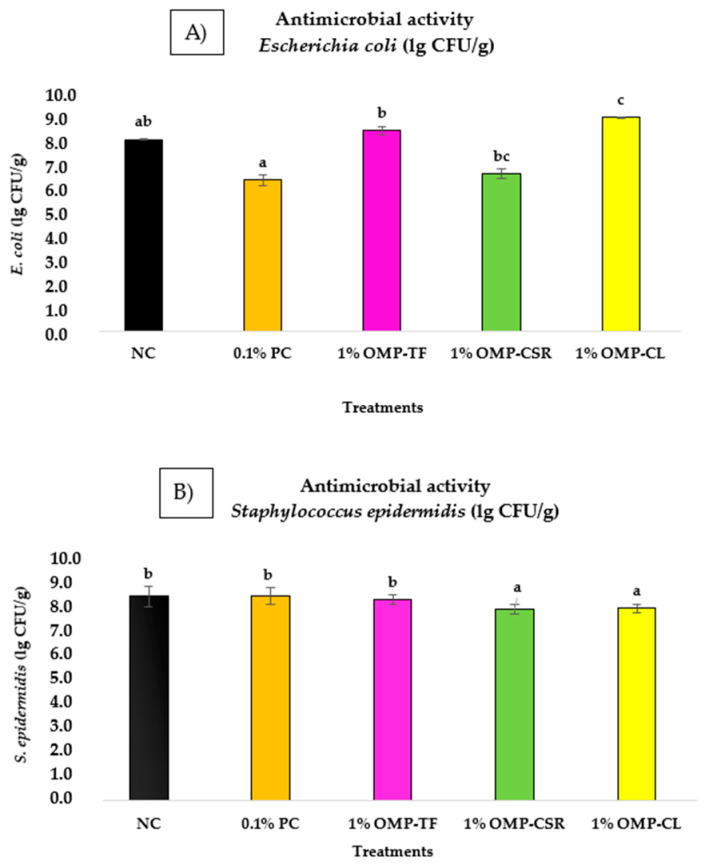
(**A**) Testing antimicrobial effect on the growth of *S. epidermidis* → (MRS liquid broth + 1% OMP-TF; 1% OMP-CSR; 1% OMP-CL) compared with control groups: (1) *S. epidermidis*+NC (MRS without added supplementation) and (2) 0.1% PC (MRS + 0.1% (*v*/*v*) β-glucans extracted from *S. cerevisiae* (Medinvest Hungary Ltd.)) were tested (**B**) to determine their effects against *E. coli* bacteria (same treatments as A). Results are expressed as lg CFU/g. Values are presented as means ± SD (n = 6 per sample). Significance differences (*p* < 0.05) are illustrated as different alphabets (a, b).

Significant differences (*p* < 0.05) among the five groups for *S. epidermidis* and *E. coli* bacteria were demonstrated by the Kruskal–Wallis test. The bar chart below (Figure 8) illustrates the average growth of tested bacteria (*E. coli* (Figure 8A) and *S. epidermidis* (Figure 8B) of OMPs in the case of different treatments.

The *E. coli* count (lg CFU/g) was notably lower (*p* < 0.001) in the 0.1% PC group compared to the 1% OMP-CL group, while the 0.1% PC and NC did not differ significantly. This means that the 0.1% PC has no significant effect on bacterial growth compared with the unsupplemented group (NC). Still, it should be mentioned that the 0.1% PC treatment showed the lowest bacterial growth, with a bacterial count of 6.43 ± 0.23 lg CFU/g, which was notably lower than that of 1% OMP-CL (*p* < 0.001). Let us compare the positive control (0.1% PC) group with the OMPs. The results show significant effectiveness (*p* < 0.05) for the 0.1% PC in reducing bacterial counts compared to the mushroom-derived treatments, for instance, in the case of 1% OMP-CL (9.12 ± 0.03 lg CFU/g), 1% OMP-TF (8.53 ± 0.19 lg CFU/g), and 1% OMP-CSR (6.69 ± 0.21 lg CFU/g). However, the statistics cannot prove the efficiency of the 0.1% PC compared with the NC.

The growth of *S. epidermidis* was significantly lower (*p* < 0.05) in the 1% OMP-CSR and OMP-CL group compared to the 1% OMP-TF (7.99 ± 0.18 lg CFU/g), NC (8.13 ± 0.40 lg CFU/g), and 0.1% PC groups (8.16 ± 0.33 lg CFU/g). In the case of the cooked samples, OMP-CSR showed the greatest growth inhibition at 7.64 ± 0.19 lg CFU/g, followed by OMP-CL (7.65 ± 0.18 lg CFU/g); these groups did not differ significantly (*p* > 0.05).

## 4. Discussion

We characterized the bioactive compounds present in freeze-dried *P. ostreatus* mushroom powders produced from starter material obtained under different processing conditions using total fresh mushrooms (OMP-TF), the solid residue of cooked (90 °C, 4 h) mushrooms (OMP-CSR), and cooked (90 °C, 4 h) mushroom liquid (OMP-CL). Our findings revealed that OMP-CSR and OMP-TF might be the most effective supplements for maximizing the physiological benefits of β-glucan intake. In addition, the best antioxidant source to combat oxidative stress (polyphenols and flavonoids), with higher antioxidant activity, was achieved using OMP-CL, which is due to the unique distribution of the content of bioactive compounds (primarily water-soluble compounds) owing to its own-liquid extraction.

CNDs have been synthesized previously from several foods and food byproducts [29,30]. For example, some researchers produced CNDs from mushrooms using hydrothermal treatment [31]. However, there is no evidence of CNDs on mushrooms prepared using other gentle methods, like freeze-drying and cooking them at a low temperature (under 100 °C). Thus, we performed a long-time cooking process. It was decided to investigate the CNDs in each sample, which are water-soluble compounds; thus, the filtrate was especially interesting.

While OMP-CL contained significantly fewer glucans, polyphenols, and flavonoids than other samples (OMP-TF and OMP-CSR), it contained the highest number of carbon nanodots (CNDs) with the highest fluorescence intensity. However, the molecular weight of the carbon nanodots (CNDs) did not differ significantly between the samples, suggesting that if the same precursor was utilized, its molecular weight could remain close to that of the samples prepared using a different technology. This idea is also supported by Banger et al. (2023) [32] and Zong et al. (2024) [33].

The fluorescence intensity varied significantly, with OMP-CL displaying the highest intensity (176.72 ± 2.00 A.u.) and quantity (50 mg in 1 kg mushroom powder). These results highlight the potential of OMP-CL as an excellent source for producing high-fluorescence CNDs. According to our results for OMP-CSR (30.48 ± 0.46 A.u. with 0.86 mg CNDs in 1 kg mushroom powder), after the cooked solid fraction centrifugation, the CNDs were reduced using liquid separation. This was also supported by the data for OMP-TF (44.96 ± 7.20 A.u with 1.31 mg CNDs in 1 kg mushroom powder), which showed that the CND levels were significantly higher (*p* < 0.05) than those of OMP-CSR. It seems that the freeze-drying process supports the formation of CNDs. Still, the final concentration is very low. While liquid extraction after pre-cooking (90 °C, 4 h) the *P. ostreatus* mushroom was shown to be a valuable tool for the production of CNDs, further studies should be performed to test the case of more mushroom species and mushrooms from different flushes (distinct cycles of fruiting body production), determine these molecules’ size distribution, safety, and stability, and calculate their economic viability.

The results of the prebiotic study show that the different supplements (OMP-TF, OMP-CSL, OMP-CL) did not influence the proliferation of *L. plantarum* at the MRS agar. Meanwhile, 1% OMP-CL, with the lowest content of bioactive compounds, significantly promoted the growth of *L. casei.* While the growth of *L. casei* showed visible differences among the OMP-CL and NC treatments to the advantage of OMP-CL, the statistical analysis did not reveal significant variations in other cases or in the 0.1% PC. The higher growth stimulation activity (*L. casei*) of OMP-CL can be attributed to the fact that water-soluble compounds and CNDs could serve as a more accessible energy source for lactic acid bacteria.

Kerezoudi et al. (2021) and Alves et al. (2012) reported that *P. ostreatus* can inhibit the growth of several Gram-positive bacteria through cell wall disruption [34,35]. Furthermore, Zhao et al. (2022) reported that CNDs can be an excellent replacement for antibiotics, with their broad-spectrum activity against bacterial cells [36]. However, researchers found no evidence that CNDs are responsible for the antibacterial activity of OMPs.

Our findings suggest that pre-cooked OMP-CSR and OMP-CL samples exhibit comparable potential in inhibiting the growth of Gram-positive bacteria, particularly *S. epidermidis*. Despite OMP-CL containing significantly lower levels of β-glucans, polyphenols, and flavonoids and demonstrating weaker antioxidant activity than OMP-TF and OMP-CSR, OMP-CL had the highest concentration of CNDs. Overall, the cooked samples (OMP-CL and OMP-CSR) were effective against *S. epidermidis*. This enhanced activity may result from heat treatment disrupting fungal cell walls, thereby releasing bioactive compounds, such as phenolics, flavonoids, and polysaccharides, which are well-known for their antimicrobial properties [37]. Regarding OMPs and the positive control (0.1% PC), there were no significant positive effects (growth inhibition) against *E. coli* bacteria compared with the negative control (NC). This means that OMPs have a limited impact on Gram-negative bacteria. This can be because the presence of lipopolysaccharides (LPS) in the outer membrane of Gram-negative harmful bacteria could limit the ability of OMPs to penetrate and affect the bacteria effectively [38]; therefore, further studies should be performed, for example, applying higher concentrations (from 1.5 to 3%). In addition, the role of CND after extraction should be further investigated.

Our paper offers a new model for understanding the mechanisms of the complex matrix of bioactive compounds in antimicrobial and prebiotic functions [34,39]. Current research suggests that promoting probiotic bacterial growth and inhibiting harmful bacterial growth with cooked OMPs can be a potential alternative for achieving a healthy microbiota composition in the host’s gastrointestinal tract, thereby reducing antibiotic use and countering antibiotic resistance [40]. However, further in vitro and in vivo studies should be performed to understand the mechanisms of CNDs’ actions in various biological systems.

## 5. Conclusions and Future Perspectives

Our study underscores the significant health potential of freeze-dried *Pleurotus ostreatus* mushroom powders produced from total fresh mushrooms (OMP-TF), the solid residue of cooked (90 °C, 4 h) mushrooms (OMP-CSR), and cooked (90 °C, 4 h) mushroom liquid (OMP-CL).

OMP-CL contained the lowest amount of bioactive compounds. However, it exhibited notable antimicrobial (against *S. epidermidis*) and probiotic growth-stimulating effects *(L. casei*) at a 1 *w*/*v*% concentration. Still, it should be mentioned that there was no difference compared with the positive control (β-glucan) in a 0.1 *w*/*v*% concentration (0.1% PC). While OMP-TF contains the highest amount of bioactive compounds, it has no positive effect on probiotics and harmful bacterial growth.

While the observed glucan content, antioxidant capacity (DPPH), polyphenol and flavonoid content, and effects on probiotic growth stimulation and antimicrobial activity highlight *P. ostreatus* as a valuable resource for functional foods and supplements, several questions remain. It is unclear whether the antimicrobial and probiotic growth stimulation effect is due to the presence of carbon nanodots because mushroom powder from the cooked solid residue (OMP-CSR) also has significant antimicrobial benefits against *S. epidermidis*; therefore, this study should be supported with further evidence in the future. Do the extracted CNDs exhibit antimicrobial activity against various bacterial and fungal pathogens? What are the toxicological properties of CNDs and their broader effects on antibiotic-resistant harmful microorganisms? Can mushroom-based CNDs suppress fungal growth, expanding their applications in agriculture and medicine?

## Figures and Tables

**Table 1:** Abbreviations of mushroom powders produced using different technologies and procedure descriptions. All samples were freeze-dried (40 °C, 24 h) and ground into fine powders. Fresh samples were used for the experiments. Abbreviations: **OMP-TF**, **o**yster **m**ushroom **p**owder from the **t**otal **f**resh sample; **OMP-CSR**, **o**yster **m**ushroom **p**owder from the **c**ooked **s**olid **r**esidue; **OMP-CL**, **o**yster **m**ushroom **p**owder from the **c**ooked **l**iquid.

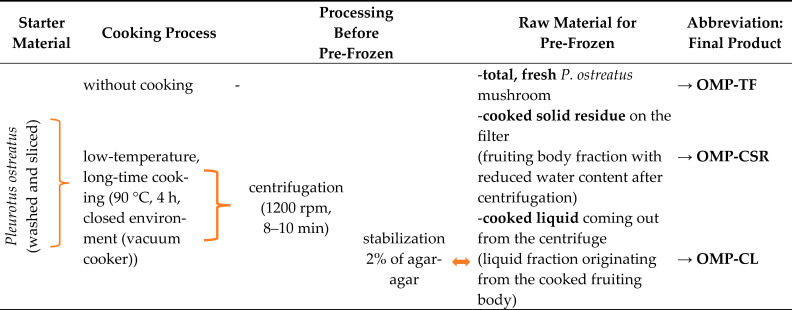

## Data Availability

The original contributions presented in the study are included in the article, further inquiries can be directed to the corresponding author.

## References

[1] Guo W., Tang X., Cui S., Zhang Q., Zhao J., Mao B., Zhang H. (2024). Recent Advance in Quality Preservation of Non-Thermal Preservation Technology of Fresh Mushroom: A Review. Crit. Rev. Food Sci. Nutr..

[2] Cerletti C., Esposito S., Iacoviello L. (2021). Edible Mushrooms and Beta-Glucans: Impact on Human Health. Nutrients.

[3] Heleno S.A., Ferreira R.C., Antonio A.L., Queiroz M.-J.R.P., Barros L., Ferreira I.C.F.R. (2015). Nutritional Value, Bioactive Compounds and Antioxidant Properties of Three Edible Mushrooms from Poland. Food Biosci..

[4] Silva M., Ramos A.C., Lidon F.J., Reboredo F.H., Gonçalves E.M. (2024). Pre- and Postharvest Strategies for Pleurotus Ostreatus Mushroom in a Circular Economy Approach. Foods.

[5] Devi P.V., Islam J., Narzary P., Sharma D., Sultana F. (2024). Bioactive Compounds, Nutraceutical Values and Its Application in Food Product Development of Oyster Mushroom. J. Future Foods.

[6] Chang R., Chen L., Qamar M., Wen Y., Li L., Zhang J., Li X., Assadpour E., Esatbeyoglu T., Kharazmi M.S. (2023). The Bioavailability, Metabolism and Microbial Modulation of Curcumin-Loaded Nanodelivery Systems. Adv. Colloid Interface Sci..

[7] Loo Y.T., Howell K., Chan M., Zhang P., Ng K. (2020). Modulation of the Human Gut Microbiota by Phenolics and Phenolic Fiber-rich Foods. Compr. Rev. Food Sci. Food Saf..

[8] Mihai R.A., Melo Heras E.J., Florescu L.I., Catana R.D. (2022). The Edible Gray Oyster Fungi *Pleurotus ostreatus* (Jacq. ex Fr.) P. Kumm a Potent Waste Consumer, a Biofriendly Species with Antioxidant Activity Depending on the Growth Substrate. J. Fungi.

[9] Assemie A., Abaya G. (2022). The Effect of Edible Mushroom on Health and Their Biochemistry. Int. J. Microbiol..

[10] Kılıç C., Gürgen A., Yıldız S., Can Z., Değirmenci A. (2024). Total Phenolics, Tannin Contents, Antioxidant Properties, Protein and Sensory Analysis of *Pleurotus ostreatus*, *Pleurotus citrinopileatus* and *Pleurotus djamor* Cultivated on Different Sawdusts. Maderas-Cienc. Tecnol..

[11] Ayser M., Tonny W., Hernandez I.S., Kuriakose R., Smith J.D., Wallaert S.J., Karim A., Robertson M.L., Balan V. (2023). Fractionating Chitin-Glucan Complex and Coproducts from Pleurotus Ostreatus Mushrooms. Waste Biomass Valor..

[12] Selli S., Guclu G., Sevindik O., Kelebek H. (2021). Variations in the Key Aroma and Phenolic Compounds of Champignon (*Agaricus bisporus*) and Oyster (*Pleurotus ostreatus*) Mushrooms after Two Cooking Treatments as Elucidated by GC–MS-O and LC-DAD-ESI-MS/MS. Food Chem..

[13] Sun Y., Lv F., Tian J., Ye X.Q., Chen J., Sun P. (2019). Domestic Cooking Methods Affect Nutrient, Phytochemicals, and Flavor Content in Mushroom Soup. Food Sci. Nutr..

[14] Murata M. (2021). Browning and Pigmentation in Food through the Maillard Reaction. Glycoconj. J..

[15] Ahmed K.J.A.J., Bornare D., Jaiswal S. (2024). Review on Extraction of Melanoidins from Coffee Waste and Value Addition in Food. J. Curr. Res. Food Sci..

[16] Prajapati B.G., Pandey V., Sharma S., Patel S., Shah D.P., Kapoor D.U. (2024). Carbon Nanodots: An Illuminating Paradigm in Production, Characterization, and Oncological Targeting Methodologies—A Review. BioNanoScience.

[17] Tejwan N., Kundu M., Ghosh N., Chatterjee S., Sharma A., Abhishek Singh T., Das J., Sil P.C. (2022). Synthesis of Green Carbon Dots as Bioimaging Agent and Drug Delivery System for Enhanced Antioxidant and Antibacterial Efficacy. Inorg. Chem. Commun..

[18] Lysenko V., Kuznietsova H., Dziubenko N., Byelinska I., Zaderko A., Lysenko T., Skryshevsky V. (2024). Application of Carbon Dots as Antibacterial Agents: A Mini Review. BioNanoScience.

[19] Izham I., Avin F., Raseetha S. (2022). Systematic Review: Heat Treatments on Phenolic Content, Antioxidant Activity, and Sensory Quality of Malaysian Mushroom: Oyster (*Pleurotus* spp.) and Black Jelly (*Auricularia* spp.). Front. Sustain. Food Syst..

[20] Shahidi F., Yeo J. (2016). Insoluble-Bound Phenolics in Food. Molecules.

[21] Tu J., Brennan M., Brennan C. (2021). An Insight into the Mechanism of Interactions between Mushroom Polysaccharides and Starch. Curr. Opin. Food Sci..

[22] Törős G., Béni Á., Peles F., Rai M., Elramady H., Prokisch J. (2024). Enhancing Biomass and &beta;-Glucan Yield from Oyster Mushroom *Pleurotus ostreatus* (Agaricomycetes) Mycelia through Extract Valorization. Int. J. Med. Mushrooms.

[23] McCleary B.V., Draga A. (2016). Measurement of β-Glucan in Mushrooms and Mycelial Products. J. AOAC Int..

[24] Srivastava M., Kumari M., Karn S.K., Bhambri A., Mahale V.G., Mahale S. (2024). Submerged Cultivation and Phytochemical Analysis of Medicinal Mushrooms (*Trametes* sp.). Front. Fungal Biol..

[25] Wickramasinghe M.A., Nadeeshani H., Sewwandi S.M., Rathnayake I., Kananke T.C., Liyanage R. (2023). Comparison of Nutritional Composition, Bioactivities, and FTIR—ATR Microstructural Properties of Commercially Grown Four Mushroom Species in Sri Lanka; Agaricus Bisporus, Pleurotus Ostreatus, *Calocybe* sp. (MK-White), Ganoderma Lucidum. Food Prod. Process Nutr..

[26] Ahmad N., Mahmood F., Khalil S.A., Zamir R., Fazal H., Abbasi B.H. (2014). Antioxidant Activity via DPPH, Gram-Positive and Gram-Negative Antimicrobial Potential in Edible Mushrooms. Toxicol. Ind. Health.

[27] Nguyen D.H.H., Muthu A., El-Ramady H., Daróczi L., Nagy L., Kéki S., Béni Á., Csarnovics I., Prokisch J. (2024). Optimization of Extraction Conditions to Synthesize Green Carbon Nanodots Using the Maillard Reaction. Mater. Adv..

[28] Hassan R.A., Shafi M.E., Attia K.M., Assar M.H. (2020). Influence of Oyster Mushroom Waste on Growth Performance, Immunity and Intestinal Morphology Compared with Antibiotics in Broiler Chickens. Front. Vet. Sci..

[29] Huang C.-C., Hung Y.-S., Weng Y.-M., Chen W., Lai Y.-S. (2019). Sustainable Development of Carbon Nanodots Technology: Natural Products as a Carbon Source and Applications to Food Safety. Trends Food Sci. Technol..

[30] Park S.Y., Lee H.U., Park E.S., Lee S.C., Lee J.-W., Jeong S.W., Kim C.H., Lee Y.-C., Huh Y.S., Lee J. (2014). Photoluminescent Green Carbon Nanodots from Food-Waste-Derived Sources: Large-Scale Synthesis, Properties, and Biomedical Applications. ACS Appl. Mater. Interfaces.

[31] Venkateswarlu S., Viswanath B., Reddy A.S., Yoon M. (2018). Fungus-Derived Photoluminescent Carbon Nanodots for Ultrasensitive Detection of Hg^2+^ Ions and Photoinduced Bactericidal Activity. Sens. Actuators B Chem..

[32] Banger A., Gautam S., Jadoun S., Jangid N.K., Srivastava A., Pulidindi I.N., Dwivedi J., Srivastava M. (2023). Synthetic Methods and Applications of Carbon Nanodots. Catalysts.

[33] Zong Q., Chen H., Zhao Y., Wang J., Wu J. (2024). Bioactive Carbon Dots for Tissue Engineering Applications. Smart Mater. Med..

[34] Kerezoudi E.N., Mitsou E.K., Gioti K., Terzi E., Avgousti I., Panagiotou A., Koutrotsios G., Zervakis G.I., Mountzouris K.C., Tenta R. (2021). Fermentation of *Pleurotus ostreatus* and *Ganoderma lucidum* Mushrooms and Their Extracts by the Gut Microbiota of Healthy and Osteopenic Women: Potential Prebiotic Effect and Impact of Mushroom Fermentation Products on Human Osteoblasts. Food Funct..

[35] Alves M., Ferreira I., Dias J., Teixeira V., Martins A., Pintado M. (2012). A Review on Antimicrobial Activity of Mushroom (Basidiomycetes) Extracts and Isolated Compounds. Planta Med..

[36] Zhao W.-B., Wang R.-T., Liu K.-K., Du M.-R., Wang Y., Wang Y.-Q., Zhou R., Liang Y.-C., Ma R.-N., Sui L.-Z. (2022). Near-Infrared Carbon Nanodots for Effective Identification and Inactivation of Gram-Positive Bacteria. Nano Res..

[37] Ahmed T., Suzauddula M., Akter K., Hossen M., Islam M.N. (2024). Green Technology for Fungal Protein Extraction—A Review. Separations.

[38] Liu M., Cheng J.-H., Zhao H., Yu C., Wu J. (2024). Targeting the Outer Membrane of Gram-Negative Foodborne Pathogens for Food Safety: Compositions, Functions, and Disruption Strategies. Crit. Rev. Food Sci. Nutr..

[39] Stojanov S., Berlec A., Štrukelj B. (2020). The Influence of Probiotics on the Firmicutes/Bacteroidetes Ratio in the Treatment of Obesity and Inflammatory Bowel Disease. Microorganisms.

[40] Solis-Cruz B., Hernandez-Patlan D., Hargis B.M., Tellez G. (2019). Use of Prebiotics as an Alternative to Antibiotic Growth Promoters in the Poultry Industry. Prebiotics and Probiotics-Potential Benefits in Nutrition and Health.

